# *De novo* assembly of seven multidrug-resistant (MDR) Staphylococci genomes from biofilms of hemodialysis tunneled cuffed catheter tips of patients undergoing renal failure treatment

**DOI:** 10.1128/mra.00435-25

**Published:** 2025-09-08

**Authors:** Rajsekhar Adhikary, Indrani Sarkar, Dhara Patel, Sishir Gang, Uttam Kumar Nath, Saugata Hazra

**Affiliations:** 1 Department of Bioscience and Bioengineering, Indian Institute of Technology-Roorkee 30112, Roorkee, Uttarakhand, India; 2 Department of Medical Oncology and Haematology, AIIMS Rishikesh 442339, Rishikesh, Uttarakhand, India; 3 Department of Medical Laboratory Technology, BapubhaiDesaibhai Patel Institue of Paramedical Sciences (BDIPS), Charotar University of Science and Technology, CHARUSAT campus 130172, Anand, Gujarat, India; 4 Department of Nephrology, Muljibhai Patel Urological Hospital 29025, Nadiad, Gujarat, India; 5 Centre for Nanotechnology, Indian Institute of Technology-Roorkee 30112, Roorkee, Uttarakhand, India; Loyola University Chicago, Chicago, Illinois, USA

**Keywords:** *Staphylococcus *spp., *de novo* genome assembly, multidrug-resistant (MDR), biofilms, hemodialysis tunneled cuffed catheter tips, renal failure patient

## Abstract

*Staphylococcus aureus* CHRFS5, *S. aureus* HL_CHRU_S18, *S. epidermidis* S48B, *S. epidermidis* HL_CHRU_S16, *S. hominis* S19, *S. haemolyticus* HL_CHRU_S79, and *S. warneri* HL_CHRU_S111 were isolated from the biofilm of catheter tip of renal failure patients. Whole genome sequencing predicted the presence of multiple antibiotic-resistant gene cassettes.

## ANNOUNCEMENT

Catheter-related bloodstream infections (CRBSIs) are significant clinical issues affecting end-stage renal failure patients, causing 4.95 million global deaths in 2019 due to healthcare-associated infections and multidrug-resistant pathogens (1–4). The seven isolates (CHRFS5, HL_CHRU_S18, S48B, HL_CHRU_S16, S19, HL_CHRU_S79, and HL_CHRU_S111) were isolated from the biofilm of internal lumen of hemodialysis tunneled cuffed catheter tips from different renal failure patients at Muljibhai Patel Urological Hospital (Institutional ethical approval No.: MPUH/EC/788/2022). The outer surface of the tip was wiped with ethanol for bacterial isolation, then split open, suspended in peptone water, and incubated overnight at 37°C for growth enrichment, and the isolates were cultured regularly on nutrient agar medium at 37°C for 24 h in aerobic condition (150 rpm) (5). For long-term storage, the stock culture was kept at −20°C in a preservation solution of 20% NB-glycerol. For identification of bacteria using 16S rRNA sequencing, genomic DNA was extracted from the regularly grown culture using the phenol-chloroform method (6), and it was qualitatively checked by 1% agarose gel electrophoresis followed by spectrophotometrically analyzed the optical density of 260/280 nm was 1.8. Targeted 16S rRNA region was amplified using 8F (5′-AGAGTTTGATCCTGGCTCAG-3′) and 1492R (5′-GGTTACCTTGTTACGACTT-3′) primers, and sequenced using Sanger’s sequencing method (5). Identified as *Staphylococcus aureus* CHRFS5, *S. aureus* HL_CHRU_S18, *S. epidermidis* S48B, *S. epidermidis* HL_CHRU_S16, *S. hominis* S19, *S. haemolyticus* HL_CHRU_S79, and HL_CHRU_*S. warneri* S111 through 16S rRNA phylogeny (7) with the closest relatives were *S. argenteus* MSHR1132(T) (FR821777), *S. argenteus* MSHR1132(T) (FR821777), *S. epidermidis* NCTC 11047 (UHDF01000003), *S. epidermidis* NCTC 11047 (UHDF01000003), *S. hominis* subsp. *hominis* DSM 20328 (X66101), *S. haemolyticus* MTCC 3383 (LILF01000056), and *S. warneri* ATCC 27836 (L37603), respectively, according to EzBioCloud database v2023.8.23. The whole genome sequencing (WGS) analysis was performed from the previously isolated genomic DNA, followed by NEBNext Ultra II DNA library prep kit for paired-end library preparation and sequenced on the Illumina NovaSeq 6000 platform (Biokart Genomic Lab, Bengaluru, Karnataka, India), generating 2 × 151-bp paired-end reads for each direction (8).

TrimGalore v0.6.10 was used to remove the adaptor and trim all paired-end reads from the fastq files, ensuring an average Phred quality score of 38 prior to assembly (9), followed by Unicycler v0.4.8 assembler (https://github.com/rrwick/Unicycler) to complete *de novo* assembly (10). NCBI Prokaryotic Genome Annotation Pipeline v6.6 was used to annotate genomic data, and output files were generated according to standard procedures (11). The assembly’s’ characteristics were detailed in Table 1. CARD v6.0.5 and ResFinder v4.7.2 databases were used to determine the genes for antibiotic resistance (12, 13). Unless otherwise noted, all software was run with default settings.

**TABLE 1:** Genome and antibiotic resistance properties of seven pathogenic *Staphylococcus* spp.

Parameters		Genome characteristics and isolate details						
		CHRFS5	HL_CHRU_S18	S48B	HL_CHRU_S16	S19	HL_CHRU_S79	HL_CHRU_S111
Genome characters	Total length (bp)	2,794,792	2,773,305	2,427,286	2,247,243	2,207,001	2,482,403	2,610,859
	No. of reads (M)	10.8	4.6	10.2	1.0	7.0	3.4	6.8
	GC%	32.5	32.5	32.0	32.5	31.0	32.5	32.5
	Genome coverage	99.28×	97×	96.8×	96×	95.63×	95.63×	98x
	Genome completeness (%)	98.84	98.36	99.52	98.35	91.76	99.25	99.03
	N_50_ (kb)	150.5	538.5 kb	154.7	96.4	442.4	57.4	199.2
	No. of contigs	59	74	49	56	25	91	43
	No. of total Coding sequence (CDSs)	2737	2784	2264	2075	2152	2442	2577
	No. of rRNA	4	4	4	16	4	3	4
	No. of tRNA	56	56	53	80	59	57	60
	No. of ncRNA	4	4	4	4	4	4	4
	No. of plasmids	3	0	6	0	1	2	3
Antibiotic resistance genes		*mepR, mgrA, arlS, arlR, norA, norC, blaZ, vanT, kdpD, LmrS, sepA, sdrM, parC, aac(6')-aph(2''), mecA, bleO*.	*mepR, mgrA, norA, FosB,* *arlR, norC, blaZ, vanT, kdpD,* *LmrS, sepA, sdrM, gyrA, parC*	*dfrC, dfrG, mecA, FosBx1,* *norC, sdrM, sepA, vanT, blaZ,* *norA, ErmC, gyrB, mecA,* *erm(C),* *dfrG*.	*FosBx1, norC, sdrM,* *sepA, vanT, dfrC, norA*	*fusB, msrA, blaZ, sdrM, sepA, vanT, vanY, fusB*	*FosD, cfrA, mecA, vanY, norC,* *sdrM, sepA, vanT, msrA, mphC,* *qacJ,* *fexB,* AAC(6')-Ie-APH(2'')-Ia, *qacJ,* *qacG, fosD, mph(C), cfr*	*vanT, sepA, sdrM, vanW, vanY, blaZ, msrA, gyrB*
Data availability	GenBank accession	JAWDKH000000000	JBMIRJ000000000	JAWDKJ000000000	JBMIRI010000000	JAWDKI000000000	JBMIRH000000000	JBMIRK000000000
	SRA accession	SRR32895883	SRR32895886	SRR32895881	SRR32895887	SRR32895882	SRR32895885	SRR32895884

Genome analysis had predicted the predominant presence of beta-lactamase *blaZ*, penicillin binding protein 2a (PBP2a) encoding gene *mecA*, vancomycin resistant genes (*vanT, vanY, vanW*), fluoroquinolone-resistant (*dfrC, dfrG*), and other antibiotic-resistant genes (*gyrB, FosB, FosBx1*), alongside antibiotic efflux pumps and transporters (*mgrA, mepR, norC, kdpD),* extracellular protein secretion system (*SepA*) and others contributing to the development of multidrug resistance of Staphylococci (Table 1). Further mining of these pathogenic, multidrug-resistant Staphylococci genomes will explore the mechanism of virulence and antibiotic resistance emergence.

## Data Availability

The BioProject and BioSample accession numbers of the complete genomes are PRJNA1023153 and SAMN37641399, SAMN47519549, SAMN37654260, SAMN47519550, SAMN37653081, SAMN47519544, SAMN47519546, for *Staphylococcus aureus* CHRFS5, *S. aureus* HL_CHRU_S18, *S. epidermidis* S48B, *S. epidermidis* HL_CHRU_S16, *S. hominis* S19, *S. haemolyticus* HL_CHRU_S79, and HL_CHRU_*S. warneri* S111, respectively. The NCBI SRA accession numbers are SRR32895883, SRR32895886, SRR32895881, SRR32895887, SRR32895882, SRR32895885, SRR32895884 and the GenBank accession numbers JAWDKH000000000, JBMIRJ000000000, JAWDKJ000000000, JBMIRI000000000, JAWDKI000000000, JBMIRH000000000, JBMIRH000000000 for *Staphylococcus aureus* CHRFS5, *S. aureus* HL_CHRU_S18, *S. epidermidis* S48B, *S. epidermidis* HL_CHRU_S16, *S. hominis* S19, *S. haemolyticus* HL_CHRU_S79, and HL_CHRU_*S. warneri* S111, respectively.
